# Gap between contact and content in maternal and newborn care: An analysis of data from 20 countries in sub–Saharan Africa

**DOI:** 10.7189/jogh.07.020501

**Published:** 2017-12

**Authors:** Liliana Carvajal–Aguirre, Agbessi Amouzou, Vrinda Mehra, Meng Ziqi, Nabila Zaka, Holly Newby

**Affiliations:** 1 Data and Analytics, Division of Data, Research and Policy, UNICEF, New York, New York, USA; 2 Institute for International Programs, Department of International Health, Johns Hopkins Bloomberg School of Public Health, Baltimore, Maryland, USA; 3 UNICEF, Program Division, New York, New York, USA; 4 Independent consultant

## Abstract

**Background:**

Over the last decade, coverage of maternal and newborn health indicators used for global monitoring and reporting have increased substantially but reductions in maternal and neonatal mortality have remained slow. This has led to an increased recognition and concern that these standard globally agreed upon measures of antenatal care (ANC), skilled birth attendance (SBA) and postnatal care (PNC) only capture the level of contacts with the health system and provide little indication of actual content of services received by mothers and their newborns. Over this period, large household surveys have captured measures of maternal and newborn care mainly through questions assessing contacts during the antenatal, delivery and postnatal periods along with some measures of content of care. This study aims to describe the gap between contact and content –as a proxy for quality– of maternal and newborn health services by assessing level of co–coverage of ANC and PNC interventions.

**Methods:**

We used Demographic and Health Surveys (DHS) data from 20 countries between 2010 and 2015. We analysed the proportion of women with at least 1 and 4+ antenatal care visit, who received 8 interventions. We also assessed the percentage of newborns delivered with a skilled birth attendant who received 7 interventions. We ran random effect logistic regression to assess factors associated with receiving all interventions during the antenatal and postnatal period.

**Results:**

While on average 51% of women in the analysis received four ANC visits with at least one visit from a skilled health provider, only 5% of them received all 8 ANC interventions. Similarly, during the postnatal period though two–thirds (65%) of births were attended by a skilled birth attendant, only 3% of newborns received all 7 PNC interventions. The odds of receiving all ANC and PNC interventions were higher for women with higher education and higher wealth status.

**Conclusion:**

The gap between coverage and content as a proxy of quality of antenatal and postnatal care is excessively large in all countries. In order to accelerate maternal and newborn survival and achieve Sustainable Development Goals, increased efforts are needed to improve both the coverage and quality of maternal and newborn health interventions.

Over the past 25 years, concerted global efforts have led to dramatic reductions in maternal and under–five mortality. Globally, the maternal mortality ratio has declined by nearly 44%, [1] while the under–five mortality rate has fallen by 53% [2]. Yet, most low and middle income countries failed to attain the maternal, newborn and child health goals set out in the Millennium Development Goals (MDGs) [3] and an unacceptably large numbers of women, newborn and children are still dying. About 800 women and 7700 newborns die each day from complications during pregnancy and childbirth and in the postnatal period [4]. Increasing newborn survival is a continuing challenge that must be addressed as neonatal deaths are becoming an increasing share of under–five deaths. [3]. Thus, a major unfinished agenda is the annual toll of 2.9 million neonatal deaths which account for 45% of all under–five deaths [5,6]. It is now well established that care around the time of birth has the potential to avert more than 40% of neonatal deaths and must be prioritized as the world seek to eliminate preventable neonatal deaths [7]. Key proven interventions include care by a skilled birth attendant, emergency obstetric care, immediate care for every newborn baby including breastfeeding support and clean birth practices such as cord and thermal care and newborn resuscitation [2]. Evidence also suggests that increased coverage and quality of preconception, antenatal, intrapartum, and postnatal interventions by 2025 could avert 71% of neonatal deaths, 33% of stillbirths and 54% of maternal deaths per year [7].

Monitoring the coverage of effective and affordable maternal, newborn and child health interventions is central to assess progress [8,9]. For the purpose of global monitoring and reporting, a set of coverage indicators along the continuum of care have been adopted by global monitoring frameworks like the Global Strategy for Women’s, Children’s and Adolescents’ Health 2016–2030 and the Every Newborn Action Plan, to mention a few [10–12]. More women are now receiving antenatal care and delivering with a skilled attendant. Globally, antenatal care coverage for 4 or more antenatal visits by any provider has increased from 35% in 1990 to 58% in 2015 [13], while the proportion of births delivered with a skilled birth attendant rose from 61 to 78% between 1990 and 2015 [14]. However, these changes in coverage of maternal and newborn health have not reflected expected progress in impact indicators related to maternal and newborn survival. It is being increasingly recognized that the global measures of coverage of maternal and newborn health capture only contacts with the health system with little information about the quality of care received. Maximizing coverage of measures focused on contacts alone is insufficient to reduce maternal, newborn and child mortality. To move towards elimination of preventable causes of maternal and newborn deaths, increased coverage of recommended contacts should be accompanied by increased focus on content of services [4,15–21]. Recent evidence shows that closure of quality gap of facility based maternal and newborn health services could prevent an estimated 113 000 maternal deaths, 531 000 stillbirths and 1.325 million neonatal deaths annually by 2020 [7].

Currently, the global indicators specific to pregnancy, delivery and postnatal periods that are common to the Global Strategy and ENAP include antenatal care (at least four visits), skilled attendant at birth and postnatal care for mothers and newborns within 48 hours following birth. These global maternal and newborn health indicators are truly the tip of the iceberg as these focus only on contacts between women or newborns with the health system and provide no indication of the content of services and quality of care delivered, which limits their usefulness for programmatic purposes [22]. A critical gap is noted in the measurement and reporting of quality of services received by women and children with the recommendation of adding core indicators assessing quality of maternal and newborn health care to the global coverage indicators [4,12,18,23,24]. Recently, the World Health Organization has proposed standards of care and measures assessing quality of maternal and newborn health care [4].

Large–scale, nationally representative household surveys such as UNICEF–supported Multiple Indicator Cluster Surveys (MICS) [25] and USAID–supported Demographic and Health Surveys (DHS) [26] are the largest source of data on maternal and child health outcomes at the population level. But, these surveys are limited in terms of providing information on content of care during the antenatal, labour, delivery and postnatal period. Data are often collected on basic services received during antenatal care such weighing, testing of urine and blood, measuring blood pressure, tetanus protection, etc. During intra and postpartum periods, information on initiation of breastfeeding, weighing, immunization and postnatal care of mother and newborn is collected. While this information does not cover the breath of all services required, and especially in cases of emergency care and treatment, together, it can allow an assessment of whether women and newborns are receiving the minimum expected services. Thus, data collected through MICS and DHS has the potential to provide an indication of level of quality of care, at least at a basic level. Unlike health facility or quality of care surveys that focus on care provided at service delivery sites, these household surveys have the advantage to provide nationally representative estimates that can also be disaggregated by relevant background characteristics including sub–national regions, mother’s education, mother’s age, sex of the child, wealth quintiles, etc., and allow to conduct relevant equity analyses which are a priority in the Sustainable Development Goals (SDGs) era.

In this paper, we analyse the co–coverage of content interventions used as proxy for quality of care received by women during antenatal care and by the newborn during postnatal period using data from nationally representative surveys. We then compare this co–coverage estimate with the global coverage indicators assessing contacts with health system to highlight the gap between contact and content.

## METHODS

### Data Source

Data for this study are from DHS surveys conducted between 2010 and 2015. We used data on interventions during the antenatal, delivery and postnatal periods from DHS surveys in 20 countries (see Table S1 in **Online Supplementary Document**). These 20 countries were included due to the availability of data on 8 antenatal care (ANC) and 7 postnatal care (PNC) interventions included in this analysis. Of the 20 countries, 18 countries had data on the full set of ANC interventions and 17 countries reported on all 7 PNC interventions included in the analysis.

### Method of analysis

To assess the quality of maternal and newborn health services during pregnancy, birth and postnatal period, we analysed the co–coverage of selected interventions received by mothers and newborns. The co–coverage indicator, proposed in 2005, is a simple count of how many interventions are received by mothers and newborns out of a set of selected interventions [27].

For the purpose of this analysis, we included 8 ANC content interventions as a proxy for quality of antenatal care (**Table 1**). We first assessed the contact coverage estimates defined as (1) percent of women with a live birth in last 2 years who had at least one ANC visit with a skilled provider and (2) percent of women with a live birth in previous 2 years who had four or more ANC visits with at least one visit with a skilled health personnel. We then described coverage of content among all women with a live birth in previous 2 years and also restricted to women who reported having an ANC contact as the proportion of women with at least one ANC visit and those with four or more visits who received all 8 interventions.

**Table 1:** Set of interventions included for co–coverage analysis

8 interventions during antenatal period	7 interventions during postnatal period
1. Urine test	1. Newborn weighed at birth
2. Blood pressure taken	2. Early initiation of breastfeeding
3. Blood sample	3. No pre–lacteal feed during first three days of life
4. Iron supplementation	4. BCG vaccination
5. Tetanus protection	5. Polio vaccination at birth
6. Counselled on pregnancy complications	6. Postnatal care for newborn within 2 days of birth
7. Tested for HIV and received results	7. Postnatal care for the mother within 2 days of birth
8. 3 doses of Intermittent preventive treatment of malaria in pregnancy (IPTP)	

In order to compare the gap between contact and content at the time of birth we included 7 PNC interventions. Interventions as weighing the newborn at birth, early initiation of breastfeeding, vaccinating the newborn with Polio dose 0 and BCG were included as proxy for quality as these are directly within the control of the skilled birth attendant. No prelacteal feeds for first 3 days was included as educating and assisting women on initiating exclusive breastfeeding and maintaining successful breastfeeding has been identified as a core function of skilled health personnel [28]. Postnatal health checks within 48 hours of birth for the mother and newborn was included due to lack of data availability on content of postnatal care in the analysed household surveys. For PNC, we analysed women delivering with a skilled birth attendant (SBA) whose surviving newborn received the 7 interventions. In the present analysis, a skilled birth attendant was identified based on the database maintained by UNICEF and Countdown which validates the skill and qualifications of the health personnel. For postnatal interventions, data on immunization was collected only on surviving children. We therefore, restricted the analysis to surviving children under 2 years at the time of the survey. This may positively affect the results if it is assumed that children who have died may be more likely to have had low quality care.

To assess the factors associated with the receipt of all interventions during ANC and PNC periods, we carried out random effect logistic regression on pooled data on women who had a contact. The regression model controlled for several maternal, socio–demographic characteristics as maternal age, education status, parity, area of residence and wealth status.

## RESULTS

### Antenatal period

**Figure 1:**
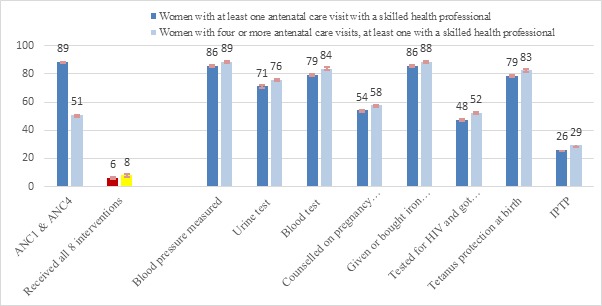
**A.** Percentage of women with a live birth in last 2 years receiving the complete set of 8 antenatal care (ANC) interventions; average across 18 countries, Demographic and Health Surveys (DHS) 2010–2015. The analysis included 18 countries as Burundi, and Rwanda did not have information about the full set of interventions. **B.** Percentage of women with at least one ANC visit and women with four or more visits by ANC intervention received; average across 18 countries, DHS 2010–2015. The analysis included 18 countries as Burundi, and Rwanda did not have information about the full set of interventions.

The analysis presented in **Figure 1** characterizes the quality of care received, among women who reported receiving at least one ANC visit with a skilled provider and those with four or more ANC visits. The gap between contact and content, defined as the difference between the percentage with four or more antenatal care visits and the percentage who received all 8 interventions, in the antenatal period is huge; compared to an average of 51% [range: 32%–76%] of women who received four or more ANC visits with at least one visit with a skilled health provider, only 5% (range: 0.3%–19%) of the women received all 8 ANC interventions (panel A in **Figure 1**). Among all interventions provided to women who had a contact during the antenatal period, receipt of three doses of intermittent preventive treatment of malaria in pregnancy was lowest (panel B in **Figure 1**). The gap between contact and content was found to be widest in case of Congo and Gabon where difference of 70 percentage points was noted between percentage of women who received 4+ ANC and the percentage of women who received all 8 ANC content interventions (see Table S2a in **Online Supplementary Document**).

**Figure 2:**
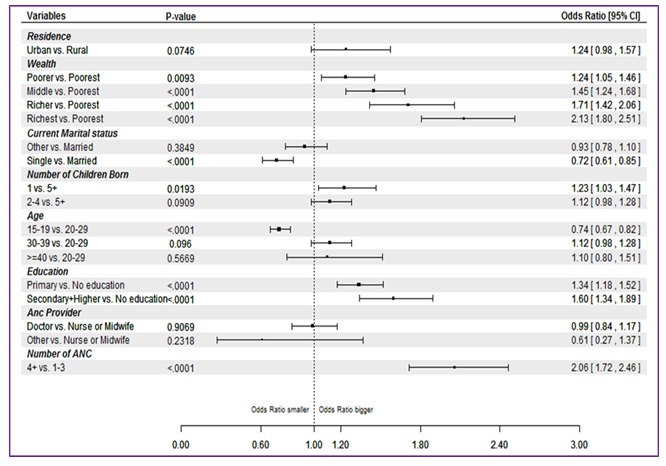
Odds ratios, 95% confidence intervals and p–values of receipt of all 8 antenatal care (ANC) interventions among women with antenatal contact from random effect logistic regression, (pooled Demographic and Health Surveys (DHS) data from 18 countries, DHS 2010–2014). The analysis included 18 countries as Burundi, and Rwanda did not have information about the full set of interventions.

The logistic regression analysis showed that women who had four or more ANC visits had 2 times higher odds of receiving all 8 interventions than those with only 1 ANC visit (odds ratio (OR) = 2.06, 95% confidence interval (CI) = 1.72–2.46). It was also found that primiparous women had 23% increased odds to receive all 8 ANC interventions compared to women with 5 or more children. The odds of receiving all ANC interventions increased significantly with greater levels of education and wealth status (**Figure 2**).

### Postnatal period

**Figure 3:**
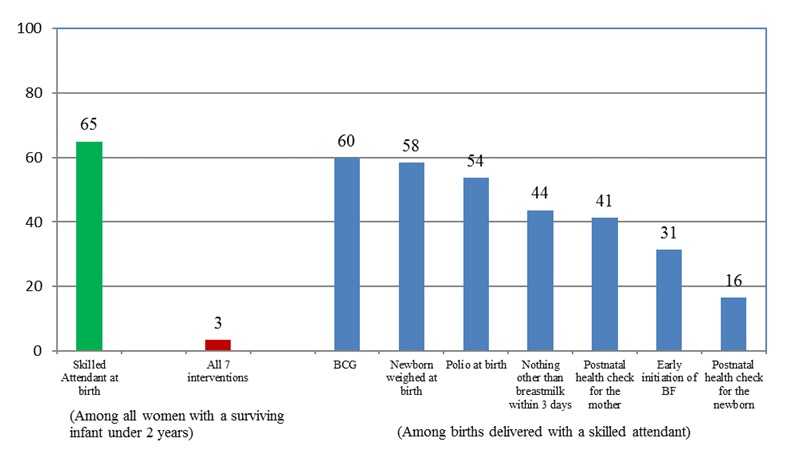
Percentage of newborns/mothers by type of intervention received during postnatal period, average across 17 countries, Demographic and Health Surveys (DHS) 2010–2015. The analysis included 17 countries as Cameroon, Mozambique and Zimbabwe did not have information about the full set of interventions.

The gap between contact and content of care highlights that though about two–thirds (65%, range: 34% to 93%) of women and newborns had contact with the health system only a handful are able to report receiving all 7 interventions considered (3%, range: <1% to 9%). (**Figure 3**). In the postnatal period, this gap was found to be the widest for Congo and Gabon. (see Table S2b in **Online Supplementary Document**).

**Figure 4:**
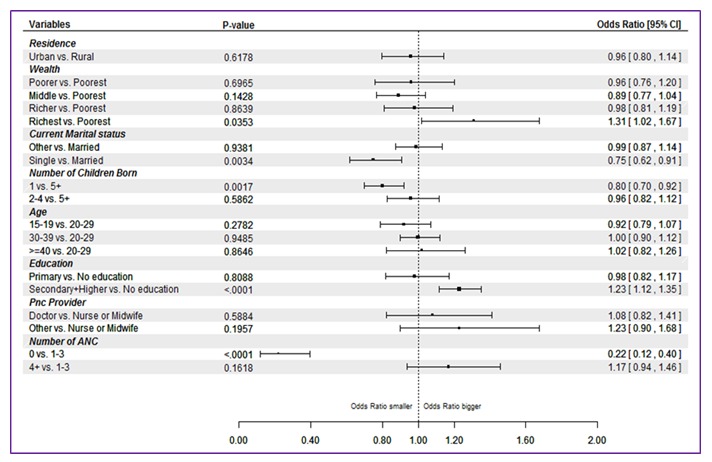
Odds ratios, 95% confidence intervals and p–values of receipt of all postnatal care (PNC) interventions from random effect logistic regression (pooled Demographic and Health Surveys (DHS) data from 17 countries, DHS 2010–2014). The analysis included 17 countries as Cameroon, Mozambique and Zimbabwe did not have information about the full set of interventions.

As with ANC interventions, the likelihood of receiving all 7 PNC interventions was higher for newborns born to women with higher education (OR = 1.23, 95% CI = 1.12–1.35) and wealth status (OR = 1.31, 95% CI = 1.02–1.67). Contact during antenatal period was also found to be associated with the receipt of PNC interventions. Analysis revealed that the odds of newborns receiving all PNC interventions were 17% (OR = 1.17, 95% CI = 0.94–1.46) more for newborns whose mothers received four or more ANC visits than those who received 1–3 visits (**Figure 4**)

## DISCUSSION

Our analysis demonstrates that there are large gaps between contact and content of care during antenatal, birth and postnatal period across all countries, as assessed using mothers’ recall from household survey. Among all ANC interventions included in the analysis, measurement of blood pressure was found to be the most commonly received intervention. Our finding resonates with an earlier study which assessed the content of antenatal care when data on antenatal interventions such as h8 and weight checking, blood pressure testing, and blood and urine testing was first available in Demographic and Health surveys [29]. The findings of the present study are also consistent with other studies that examined coverage of high quality contacts during the antenatal and postnatal period [24,29–31]. A recent study noted a substantial decline in the coverage of at least one antenatal contact and skilled birth attendance on adding content in Nigeria, Ethiopia and India [30]. Such gaps between globally recommended coverage indicators measuring contacts and actual content indicate ineffective care resulting in lack of accelerated progress towards maternal and newborn survival.

A limitation of this analysis is that we were able to analyse only interventions that were available in household surveys across the countries included in the analysis. We recognize that the scope of essential newborn care is broader and encompasses a range of interventions. Additional essential newborn care interventions such as thermal care and cord care have recently started to be included in household surveys. However, at the time of analysis data on additional newborn care interventions was available for only a few countries. Thus, our analysis included a subset of interventions in the antenatal and postnatal period for which data were available for a larger number of countries. Another limitation is that all measures included in the analysis are based on mother’s recall of care during the antenatal and postnatal period and therefore may be subject to differential recall bias. Further, only few studies have assessed the validity of coverage indicators for MNCH interventions measured through household surveys. A recent series on “Measuring Coverage in MNCH” found that the sensitivity and specificity of coverage indicators is highly variable across interventions and women report less accurately about interventions that occurred immediately following childbirth [9].

An area of further research would be linking data from facilities surveys with population based data in order to better understand the quality of available services. Recent studies linking these two sources have found an association between service readiness in health facilities and the likelihood of receiving an appropriate set of essential newborn care interventions, as well as highlighted important gaps in service delivery as obstacles to universal access to health services [32,33]. The current global maternal, newborn and child health coverage indicators for pregnancy, labour and postnatal period focus merely on contacts with the health system with no information on quality and process of care. These measures of MNCH coverage only show whether services are reaching intended beneficiaries but do not assess the effectiveness or actual content of the care received. Our analysis establishes that focusing on merely contacts with health system rather than on content of care is a critical gap in assessing the true effectiveness of maternal and child health interventions. For example, we observed that although 2 in 3 births were attended by a skilled birth attendant, only 3% of the births received all 7 interventions recommended during the immediate postnatal period.

There is increasing evidence to support that increased coverage of recommended contacts alone is insufficient to reduce maternal and neonatal mortality and morbidity [4,7,15–21,24]. Quality of care is being internationally recognized as a critical aspect of the unfinished maternal and newborn health agenda [4,15]. Our findings also highlight the need to include elements of quality of care for regular monitoring through health management information systems (HMIS), household and facility surveys in other to identify the real gaps in effective coverage. Periodic program assessments can include a measure for content analysis of ANC and PNC visits in a given sample of mothers and newborns and explore reasons of omitting certain interventions which can vary from lack of competency to stock–outs of urine and haemoglobin test kits. Further research is also required to identify more sensitive indicators on quality of care and including these in future household surveys.
